# A Review of the Mechanical Properties of Graphene Aerogel Materials: Experimental Measurements and Computer Simulations

**DOI:** 10.3390/ma16051800

**Published:** 2023-02-22

**Authors:** Penghao Qi, Hanxing Zhu, Feodor Borodich, Qing Peng

**Affiliations:** 1 School of Engineering, Cardiff University, Cardiff CF24 3AA, UK; 2 College of Aerospace Engineering, Chongqing University, Chongqing 400044, China; 3 State Key Laboratory of Nonlinear Mechanics, Institute of Mechanics, Chinese Academy of Sciences, Beijing 100190, China

**Keywords:** aerogel, graphene, mechanical properties, computational simulation, experiment measurements

## Abstract

Graphene aerogels (GAs) combine the unique properties of two-dimensional graphene with the structural characteristics of microscale porous materials, exhibiting ultralight, ultra-strength, and ultra-tough properties. GAs are a type of promising carbon-based metamaterials suitable for harsh environments in aerospace, military, and energy-related fields. However, there are still some challenges in the application of graphene aerogel (GA) materials, which requires an in-depth understanding of the mechanical properties of GAs and the associated enhancement mechanisms. This review first presents experimental research works related to the mechanical properties of GAs in recent years and identifies the key parameters that dominate the mechanical properties of GAs in different situations. Then, simulation works on the mechanical properties of GAs are reviewed, the deformation mechanisms are discussed, and the advantages and limitations are summarized. Finally, an outlook on the potential directions and main challenges is provided for future studies in the mechanical properties of GA materials.

## 1. Introduction

Graphene has many unique and excellent properties, such as superior electrical properties, high mechanical strength, flexibility, thermal conductivity, stability, and efficient energy absorption capacity [1,2,3]. These properties have made graphene a promising candidate for a broad range of applications in many fields. However, it is difficult to obtain large intact graphene sheets in practical preparations due to the defects of graphene [4,5].

The assembly of two-dimensional graphene sheets to form GAs with three-dimensional (3D) porous structures can be a good strategy. Inheriting the unique properties of graphene and the special porous structure of aerogels, this type of material exhibits ultra-low density, high porosity, high specific surface area, compressibility, super-elasticity, and high stability [6,7,8,9,10,11,12,13,14], and has therefore attracted widespread attention. Benefiting from these properties, GAs have promising future in applications like sensors [15,16,17], capacitors [18,19], electromagnetic shields [20,21], microwave absorption [22,23,24], oil adsorbents [25,26], insulation materials [27,28]. However, the complex application conditions place high demands on the mechanical properties and structural characteristics of GAs. Therefore, in the last decade, researchers have made great efforts to prepare GA materials that can meet the demands of industrialization. In terms of preparation processes, researchers have tried freeze casting [29,30], chemical vapor deposition (CVD) [31,32], hydrothermal methods [33,34], 3D printing methods [21,35,36], and other assembly strategies [37,38] to diversify the mechanical properties of GAs. Besides, experimental observations have also been made to analyze the various factors affecting the properties of these materials. The relationships between the microstructure of the GAs and their properties are investigated by means of microscopic characterization and relevant mechanical tests [39,40,41]. Some mechanical behaviors and deformation mechanisms of GAs have been reasonably speculated and analyzed with these experimental data.

In addition, because of the rapid development of the computing power of computers and related theoretical advances, researchers have also used computer simulation methods to study GAs with complex and stochastic microstructures. Through simulation approaches, especially the molecular dynamics (MD) methods, it is possible to capture data and information that are difficult to obtain experimentally, such as deformation trends in microstructures and some microscale parameters [42,43]. Moreover, some mechanistic problems such as elastic deformation and energy dissipation are visually verified by simulation models, and some peculiar phenomena that are difficult to explain in experiments are also effectively explained by simulation models.

This review focuses on recent advances in both experimental and simulation studies of the mechanical properties of GAs. Section 2 presents research works and findings related to the parameters influencing the material properties of GAs in experimental studies. The influencing factors are summarized and discussed in terms of the essential characteristics of the building block and the structural characteristics of the GAs materials. Then, in Section 3, the simulation tools, methods, and results on the mechanical properties of GAs are analyzed, and the advantages and disadvantages of the computational models are concluded. Finally, an outlook on the potential directions and possible main challenges is provided for future studies in the mechanical properties of GAs.

## 2. Experimental Measurements

GAs are promising porous materials, however, there are still some challenges in the widespread use of this type of material. The mechanical properties of GAs are one of the main reasons that limit their applications. Therefore, it is necessary to analyze and identify the main factors to improve the mechanical properties of GAs, such as strength, stiffness, large deformation and recovery, energy absorption capacity, and fatigue resistance. It will help researchers to identify appropriate optimization approaches of GAs and improve the synthesis strategies of high-performance graphene-based aerogel materials. 

### 2.1. Production Methodologies of Graphene Aerogels

Firstly, a summary of the existing production strategies of GAs is needed. The general strategy of GAs preparation is to use graphene oxide (GO) flakes as precursors to form a 3D network structure connected by multiple intermolecular forces, such as π-π bonds, van der Waals (vdW) force, and cross-link [44,45,46] 

Over almost the past 10 years, researchers have continuously improved the production strategies for GA materials and developed new synthesis methods [47,48,49]. Currently, GA preparation methods are classified into template-free assembly, template-assisted assembly processes, and 3D printing techniques. The template-free method is widely used in practice because of the simplicity and low cost of the synthesis process. The basic synthesis mechanism of template-free methods is to enhance the attraction or reduce the repulsion between adjacent graphene sheets by reducing graphene oxide or the introduction of crosslinks. The graphene sheets can then be assembled into porous 3D structures with the help of inherently ordered stacking behavior. The more common template-free assembly methods are the hydrothermal reduction method [50], the chemical reduction method [51], and cross-linking method [52]. 

In contrast, the template assembly methods, including chemical vapor deposition (CVD) and freeze casting methods, have complex production processes and high costs, making it difficult to develop large-scale preparations. However, this type of method can artificially regulate the microstructure building process based on different templates and is one of the mainstream methods to prepare 3D GAs with ordered and layered structures [53,54]. The 3D printing method is another method for preparing ordered structures of aerogel materials [44,55,56]. This method is characterized by the ability to manipulate the GA structures at different scales, making the production of GAs highly controllable and scalable. 

We have summarized the synthesis methods and the experimentally measured mechanical properties of GA materials in the relevant literature in recent years, as shown in Table 1. It can be found that there seems to be no significant correlation between the mechanical properties of GAs and the preparation methods. The density and mechanical properties of GAs prepared by the same method can also vary considerably, while some GAs have similar properties despite the use of different production methods. This is because the main factors affecting the mechanical performance of GAs are their microstructural characteristics and some structural parameters. These characteristics of the GA materials vary greatly among the different literature. It is therefore necessary to summarize and analyze the microscale parameters that affect the mechanical performance of GAs in detail.

### 2.2. Intrinsic Characteristics of Components

GAs have a special network structure that is built by stacking graphene sheets compared to other aerogels. Thus, the intrinsic properties of the graphene sheets determine the macroscopic mechanical properties of GAs to a large extent. 

**Figure 1:**
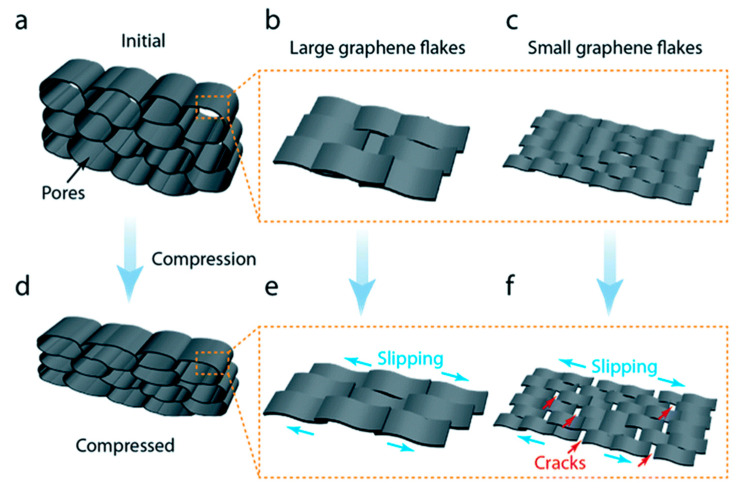
Schematics showing the deformation mechanisms of a single aerogel wall composed of graphene flakes with different sizes during compression [30]. (**a**–**c**) Larger graphene flakes has stronger interaction with adjacent flakes to resist against deformation, usually resulting in ‘slipping’. Besides ‘slipping’, (**d**–**f**) smaller flakes are easy to crack as they have weaker inter-flake conjunction.

The size scale of the graphene sheets is one of the important characteristics of GAs and can obviously affect the mechanical behaviors of this type of material [70]. Ni et al. [71] investigated the size effects of graphene sheets on the properties of GA materials. They prepared two GA samples consisting of graphene flakes of different sizes (2 μm and 20 μm) by using the freeze-casting technique. The effects of microstructure on the properties of GAs were minimized by adjusting the process parameters of the two samples to have a similar microstructure and internal morphology. After comparison, it was found that the mechanical properties of the GA constructed from small-sized (approx. 2 μm) graphene sheets were poor. By observing the microscopic image of this sample, they argued that small-sized flakes have difficulties forming a continuous tight wall structure on a large scale, and many overlapping joints and defects appear on the wall surface as a result. This ultimately causes a reduction in the mechanical properties of the cell wall of the GA, which is reflected in the poorer compressive modulus and yield stress of the macroscopic material. The GA constructed from large-size (approx. 20 μm) graphene sheets showed excellent elasticity and could recover to the original state after 80% compression strain. This mechanical behavior can be attributed to the stronger hindering effect between large-sized graphene flakes. A similar conclusion was confirmed in the studies of Wu et al. [10] and Gao et al. [30] in which the use of large graphene sheets enhances the mechanical robustness and reversible large-strain elasticity of GAs. The deformation mechanisms concluded by Gao et al. are shown in Figure 1.

In addition to the sheet size, the residual oxygen-containing functional groups in GAs are also an important factor in determining the mechanical properties of GAs. These weak interactions are derived from the precursor GO in the preparation of GAs, which give GO excellent dispersibility. However, these residual oxygen-containing functional groups can weaken the interactions between the graphene flakes in GAs, thus reducing the structural stability of this type of material [72]. Therefore, trying to remove the oxygen-containing functional groups from the base material in GAs is an effective means of obtaining high-performance GA materials. High-temperature annealing is a relatively simple treatment. Li et al. [61] prepared a GA material that has been annealed. With the help of X-ray Diffraction, they found that the proportion of oxygen-containing groups in the GA was significantly reduced, while at the same time, the elasticity of the GA was significantly improved. Qiu et al. [73] proposed a simple annealing treatment strategy that can reduce the proportion of oxygen-containing groups and the layer spacing between adjacent sheets. The GA materials with lower density and higher mechanical properties were obtained as a result. 

Šilhavík et al. [65] found that suitable high-temperature annealing treatment results in the formation of covalently cross-linked aerogel materials based on previous research [74,75] related to 3D carbon network reinforcement. They reasonably speculated that high temperature breaks the C-O bonds in the oxygen-containing groups and forms stronger C-C bond connections between the graphene sheets. This covalent bonding hinders the creation of material defects and thus produces stronger tensile and compressive strengths in GAs compared to non-covalently bonded aerogels.

Besides, the drying method during the freeze-casting process can also influence the proportion of residual oxygen-containing functional groups in GAs. Xie et al. [40,76] explored the effects of traditional freeze-drying (FD) and supercritical CO_2_ drying (ScD) on the properties and structures of GAs. They applied some surface analysis instruments and found that the ScD-processed samples have fewer residual oxygen-containing groups. In addition to a similar specific modulus, the samples processed by ScD have a lower density, a higher reversible strain (94%), and a higher corresponding stress (>160 kPa).

### 2.3. Structural Characteristics

#### 2.3.1. Microstructural Parameters

The structural features are another key factor influencing the mechanical properties of ultralight porous materials. The control of microstructural characteristics, such as the pore size and the thickness of the wall structure, is critically important for the mechanical behavior of graphene porous networks. Previous studies [77,78] have shown that the relative density and modulus of cellular materials depend on cell wall thickness and cell size. Therefore, when the density of a porous material is significantly reduced, the mechanical properties of the material are also reduced. This conclusion also applies to GA materials. Han et al. [79] and Tang et al. [80] both obtained GA materials with adjusted densities by treating the materials in certain ways. In both works, the elastic properties and strengths of the materials showed a tendency to increase with increasing density. 

**Figure 2:**
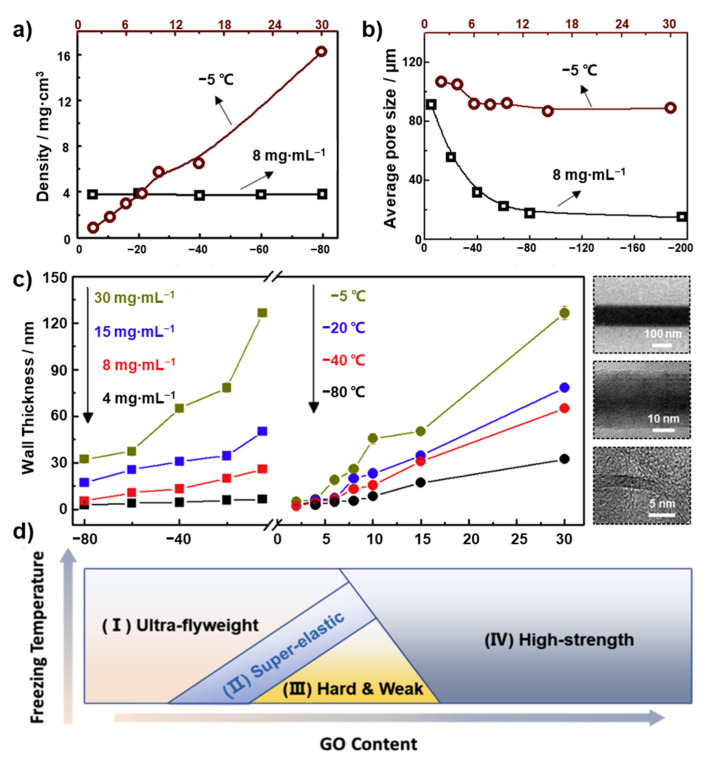
Variations in the (**a**) density and (**b**) average pore size of GAs as a function of the freezing temperature and the GO content. (**c**) Effects of the freezing temperature and GO content on the GA wall thickness. (**d**) Schematic representation of the effects of GO concentration and freezing temperature on the mechanical properties of GAs [81].

In the work of Liu et al. [81], the influences of these parameters on GA were investigated in detail. They controlled the structural parameters of the target materials, such as the wall thickness and the pore size, by adjusting the GO content and refrigerant temperature in the freeze-drying method to analyze the effects of these features on the mechanical properties of the materials, as shown in Figure 2a–c. They found that the relatively denser GA possesses a better elasticity. After extensive experimental tests, the relationships between the properties and structural parameters were concluded. The elastic modulus of the GAs is proportional to the square of their density and negatively related to their pore size. In general, the compressive stress increases with the compressive strain, and the compressive strain can be largely recovered after large deformation. They summarized the effects of the GO content and the freezing temperature on the properties of the GAs in Figure 2d [81]. Four regions were split out in this figure, the GAs in region (Ⅰ) have low density and thin walls due to low GO content during the synthesis process. As a result, the thin and fragile aerogel structures in this region have poor mechanical properties. In region (Ⅱ), the GAs possess super-elastic properties due to the reinforcement of π-π bonds between GO sheets. The GAs in region (Ⅲ) have a high wall thickness and a small pore size. Therefore, the over-stacked GO layers result in the GAs with poor elasticity and recovery, but with a high modulus to resist deformation. In region (Ⅳ), the GAs with proper pore size and wall thickness shows the highest strength and modulus. 

#### 2.3.2. Biomimetic Structure 

In the last two decades, the concept of biomimetic materials has gained a lot of attention and applied in many areas. Some exotic properties of biomimetic materials resulted from the microstructural characteristics of the materials rather than from their base material. Therefore, simulating some special structures in nature with excellent mechanical properties can also be effective in improving the properties of the materials.

The honeycomb structure is one of the natural structures to be widely studied and applied. The structure with high porosity, low density, and high mechanical properties has a good fit with aerogel metamaterials. The superior mechanical properties of low-density materials have been confirmed in previous works [82,83,84]. Therefore, Qiu et al. [6] synthesized a honeycomb-like GA material based on a bionic concept, using a controlled freeze-casting method. The high elasticity of this material was observed under compression tests. Under 80% compressive strain, the sample can withstand a weight of 5000 times its mass without collapsing. Furthermore, after one thousand cycles of compression, the material maintains a recovery ratio of 93% and retains 76% stress. Yeo et al. [39,49] applied a hierarchical design strategy to prepare an ordered structure of closed-hole GA material. The material shows excellent mechanical properties in mechanical tests because of its rhombic dodecahedral honeycomb cell structure, such as high Young’s modulus (>300 kPa) and high elasticity. 

**Figure 3:**
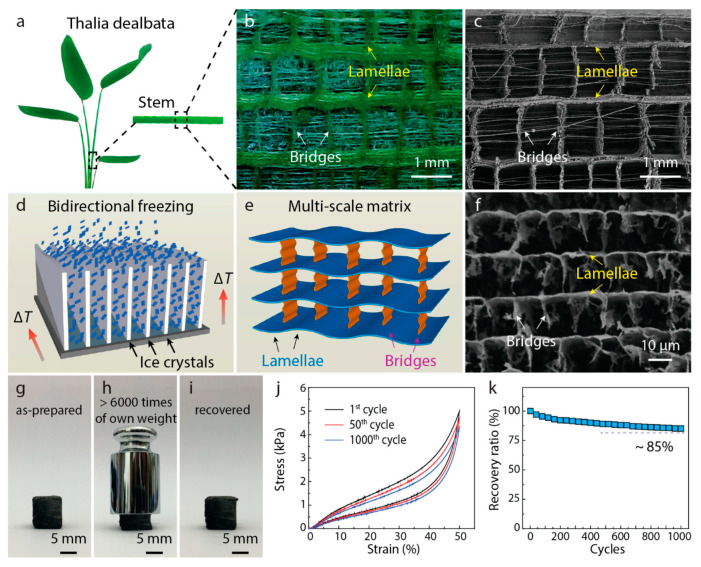
The bionic structure and mechanical tests of a biomimetic GA [12]. (**a**) Optical image of a Thalia dealbata stem. (**b**,**c**) Optical and SEM images showing the multiscale architecture, where oriented lamellar layers (thickness: ∼100 μm) are connected by interlayer bridges (length: ∼1 mm). (**d**) Mechanisms for the preparation of Thalia dealbata stem structures by bidirectional freezing techniques. (**e**) The schematic of the as-prepared graphene aerogel with plant stem-like architecture. (**f**) SEM image showing the detailed architecture of the biomimetic graphene aerogel. (**g**) Optical images showing a cubic aerogel sample (10 × 10 × 10 mm) (**h**) which supports >6000 times of its own weight with around 50% strain. (**i**) Full recovery with no obvious permanent deformation when unloading. (**j**) Representative stress–strain curves and (**k**) strength recovery ratio of an aerogel compressed (strain = 50%) and recovered after 1000 cycles.

Plant structural bionics is also a hot topic for biomimetic structure research, and some plants, such as bamboo, have excellent mechanical properties. Wegst et al. [85] provided a systematic review and commentary on the previous studies of bionic structural materials, which fully illustrates the possibilities of bionic structures in material design. Inspired by the Thalia dealbata stem, Yang et al. [12] prepared a biomimetic GA by using a template-based bi-directional freezing technology, as shown in Figure 3a–f. This bionic structure composed of lamellar layers and interconnected bridges gives the GA exceptional strength and elasticity. Under compressive test, a cube sample with a 10mm size length can retain high compressive strength after 1000 compression cycles and always have a recovery rate of over 85%, as shown in Figure 3g–k. 

Afroze et al. [27] were also inspired by a plant stem and created a GA material with a shell-core structure. This sample has a tightly packed core and a sparsely packed shell that could make a rational distribution of external loads. Therefore, this material shows significant compression performance, high elasticity, and excellent anti-fatigue properties. Recently, He et al. [53] proposed a dual template method to construct GA with a flexible structure. They produced two mimetic structural GAs by using two freezing methods and compared the deformation behavior of the two samples. GA with a bamboo-like network structure has similar mechanical properties and its elastic properties in the axial direction are two times those in the radial direction. The mechanical advantages of the bamboo-like structure are also proved in the work of Gao et al. [86]. The GA with a bamboo-like structure can be recovered to its original condition after 10 compression cycles of 80% compressive strain, and the corresponding stresses are approximately 60 kPa.

#### 2.3.3. Structures Based on Mathematical Curves and Shapes

**Figure 4:**
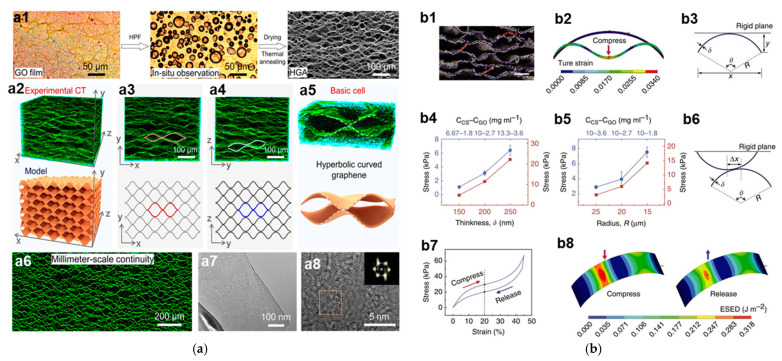
(**a**) Morphological and physical properties of hyperbolic graphene aerogel (HGA) [87]; (**a1**) In situ optical observations of ultrathin GO film during hydroplastic foaming and the SEM image of obtained HGA. (**a2**) Nano-CT image and schematic diagram of HGAs. (**a3**) XY- plane slicing images of HGAs. (**a5**) YZ-plane slicing images of HGAs. (**a4**) Nano-CT image and schematic diagram of the basic hyperbolic cell. (**a6**) Cross-sectional morphology of HGAs. (**a7**) TEM and (**a8**) HR-TEM images of HGAs after 2800 °C heat treatment. The inset is the electron diffraction pattern of the square region. (**b**) Mechanical analyses and simulations of the GA with a laminar multi-arch structure [88]. (**b1**) Microstructure of C–G monolith, showing randomly distributed bridge ligaments (marked in red dotted lines) linking adjacent lamellas. Scale bar, 20  μm. (**b2**) The true material strain (von Mises total strain) profiles of cylindrical shell under large geometry deformation. (**b3**) Schematic cross-section view of cylindrical shell mode under compression by a rigid plane. (**b4**) Compression stresses of bulk C–G monoliths with different lamella thickness (blue) and single cylindrical thin-shell mode with different shell thickness (red). (**b5**) Compression stresses of bulk C–G monoliths with different shrinkage (blue) and single cylindrical thin-shell mode with different radius (red). CCS–CGO represents the concentration of CS and GO in the initial CS–GO composite suspensions for fabricating the C–G monoliths. (**b6**) Schematic diagram of two opposite cylindrical shells with offset distance Δx compressed by a rigid plane. (**b7**) Simulated stress–strain curve based on two opposite cylindrical shells with offset distance Δx = 0.4R in a compress-release cycle. (**b8**) The elastic strain energy density profiles of cylindrical shell when the strain equal to 20% in compression and release processes (g), respectively.

Some mathematical curves and shapes have mechanical rationality in terms of structures. Therefore, after a rational design of the material microstructure, materials with an ordered mathematical structure can lead to excellent mechanical properties. Pang et al. [51,66,87] introduced hyperbolic geometry into the structure of GAs and proposed a hydroplastic foaming method to directly assemble GO sheets into this special type of GA materials. As shown in Figure 4a, this type of GAs has a layered macrostructure, with graphene bent in an arch shape. The GAs can keep their structural integrity under compression, shear, and even extreme deformation. Good anti-fatigue properties are also confirmed by the retained stress after 10^5^ compression cycles at 90% strain. Moreover, the static compression capability of this special type of GA material is so excellent that the GAs still exhibits a complete recovery of their original size and shape after 360 h at 99% strain. In other mechanical tests, this type of GAs has also shown properties that are far superior to those of ordinary GA materials. Pang et al. [51,66,87] attributed this superior mechanical performance to the effects of the hyperbolic structure on the macrostructure. They found that hyperbolic structure was able to create a seamless connection between the graphene walls, making the highly porous GA materials more durable to various deformations. In contrast, Gao et al. [88] emphasized the strengthening mechanism of the arch structure itself in the materials.

They constructed a GA with a parallel arrangement of stacked arch-shaped microstructures based on the macroscopic arched elastic structure by a bi-directional freezing process. This GA material exhibits excellent elasticity and high compressibility properties. To study the effects of arch structure on the properties of the GA, finite element methods are adopted to investigate the relationship between its cell structure and deformation behavior, as shown in Figure 4b. The simulation results show that this arch-shaped model can withstand large deformation due to its small strain. Increasing the thickness and decreasing the cell size will give this structure a higher elastic strength, which confirms the effects of the thickness of the graphene layer and material density on the elastic properties of GAs. Compared with disordered 3D graphene architectures and cellular 3D graphene architectures, this GA material can maintain relatively high compression strength, low energy loss coefficient, and low-stress loss after 10 compression cycles. Besides, more than 60% of its original stress and 93% recovery of its original size can be retained after one thousand test cycles at 80% compressive strain. The numerous arch structures share the compression of the macroscopic material by means of elastic deformation. As a result, this microstructure gives the GA material high compression properties and fatigue resistance. In the work of Zhang et al. [59], they synthesized a GA with a macroscopic hyperbolic structure by hydrothermal and freeze-casting processing. The material has a layered honeycomb-shaped microstructure. When compressed, the GA shows a negative Poisson’s ratio that can contribute to high mechanical performance [89,90]. The evolution of microstructures of this material causes a macroscopic horizontal shrinkage and a negative Poisson’s ratio response, thereby the entire area of the material is used to withstand compressive stresses. With these deformation features, this material exhibits superior structural stability and mechanical properties. 

##### D Printing Structure

**Figure 5:**
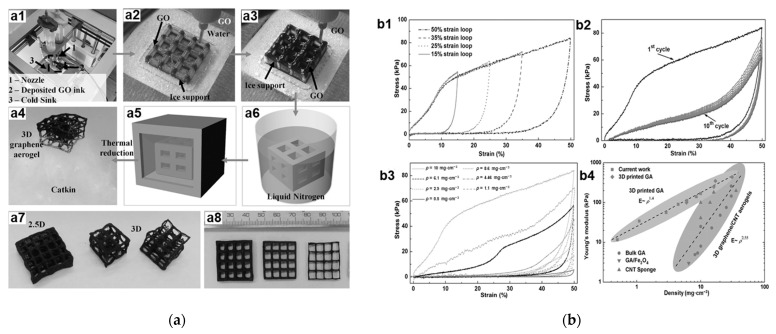
(**a**) 3D printing process and material morphology of GAs [35]; (**a1**–**a6**) 3D GA printing process; (**a7**) 3D GA architecture, left: 2.5 structure and right: 3D architecture with overhang structures. (**a8**) GAs with various wall thickness. (**b**) Mechanical property of 3D printed GAs [35]. (**b1**) Stress–strain curve during loading–unloading cycles by increasing strain amplitude for printed GA (ρ = 10 mg cm^−3^). (**b2**) The 10 cyclic loading–unloading of printed GA (ρ = 10 mg cm^−3^). (**b3**) Loading–unloading curves for printed GA with various density (from 0.5 to 10 mg cm^−3^). (**b4**) The Young’s modulus against density for existing materials.

3D bulk printing techniques can regulate the macroscopic morphology of the materials as required. Therefore, materials with excellent mechanical properties can be produced by 3D printing based on some structural optimization strategies. This method has been used to prepare high-performance materials, such as carbon composites [91], polymer foam [92], and silicone foam [93]. In contrast to conventional graphene foam structures, 3D-printed GAs generally has a periodic structure. The materials that are characterized by high surface area, high mechanical stiffness, super-elasticity, and super-compressibility depend mainly on their macrostructures. Zhu et al. [9] prepared a GA with periodic structure using a 3D printing method and GO ink deposition technology. In physical features, this material has a low density and high surface area. The elastic modulus of the GA with periodic structure is much larger than that of the bulk graphene material with a similar geometric density. Guo et al. [94] employed a special 3D printing method and prepared a lamellar GA material. Compared with the traditional GAs, this sample has good shape scalable freedom, and higher compression properties which are benefited from its small lamellar structures. This GA material can be fully recovered after a large compressive strain of 80% and a maximum compressive stress of 166.51 kPa. Zhang et al. [35] used the drop 3D printing and freeze casting processes to prepare an ultralight GA material with truss structures, as shown in Figure 5a. The rods of macroscopic truss structures are mainly subjected to tension or pressure. This method can fully utilize the material and thus reduce the weight of the structure while maintaining its mechanical performance. Therefore, the GA truss material has a low density and high mechanical properties as shown in Figure 5. 

There have been numerous studies on GA materials in recent years, but less research has been done on their mechanical properties. Although some experimental studies have pointed out some factors that affect the material properties, the actual mechanisms by which the microstructure affects the material are not clear, and the information on the deformation trend of the microstructure is difficult to capture and measure with the available instruments. Furthermore, due to the difficulty and high cost of preparing such experimental materials, the optimization of the material structures also requires simulation tools to aid the study to reduce time and material costs.

## 3. Simulation Tests

The mechanical properties of GA materials have been well characterized under extensive experimental studies in recent years. However, experimentally measured properties of GAs cannot be consistently maintained within the range required for industrialization. For example, the experimental measurements of the stiffness and strength of GAs can vary widely [89,90,91]. This is mainly due to some complex factors, such as the unstable quality of the GA samples in experiments or experimental errors. Furthermore, due to the complex structure and deformation behavior of graphene foams, their geometrical characteristics, structure cross-linking, and microstructural evolution are difficult to accurately measure and monitor through existing experimental techniques. Therefore, numerical methods and computer simulations to evaluate the constitutive relationship and the corresponding intrinsic micro/nanostructural mechanisms of the mechanical behaviors of GAs are still the best choices today. Currently, most of the research studies utilize the MD approaches for relevant mechanisms and properties of GAs. 

### 3.1. Molecular Dynamic Simulations

Molecular Dynamics (MD) method is a set of molecular simulation methods which rely on the classical Newton mechanical model to simulate the motion of molecular systems [95,96]. In this simulation system, the trajectories of particles are calculated by numerically solving motion equations of interacting particles. The state of the motion of atoms and molecules is often determined by the set interatomic potentials or molecular mechanics forcefields. The method is applied widely in molecular biology, chemical physics, and materials science as a powerful tool for the mechanistic study of objective phenomena at the molecular level [97].

#### 3.1.1. All-Atom Molecular Dynamics Simulation

All-Atom Molecular Dynamics (AAMD) method is one of the first MD modeling strategies used in the study of GAs due to its wide applicability. In atomistic simulations, the interatomic potential is the most important parameter in an MD system. In general, the interatomic potentials that need to be considered in an MD system of GAs include the carbon-carbon potentials ($E_{C-C}$), carbon-water potential ($E_{C-water}$), and water-water potential (*E_water − water_*). Therefore, the total potential energy of the MD system can be described as:

(1) $$E=E_{C-C}+E_{C-water}+E_{water-water}$$

where $E_{C-C}$ is the short-range contribution that can be described by the reactive empirical bond order (REBO) potential and torsion potential, $E_{C-water}$ and $E_{water-water}$ are both long-range contributions that are described by the Lennard-Jones (LJ) potential [98,99]. The current literature utilizes the Adaptive Intermolecular Reactive Empirical Bond-Order (AIREBO) potential from the study by Stuart et al. [100] to express the total energy of the MD system. The AIREBO potential is constituted by three terms of the REBO, torsion, and LJ potentials, so the total energy can also be described as:

(2) $$E=\frac{1}{2}\underset{i}{\sum }\underset{j\neq i}{\sum }\left[\;E_{ij}^{REBO}+E_{ij}^{LJ}+\underset{k\neq i,j}{\sum }\underset{l\neq i,j,k}{\sum }E_{kijl}^{Torsion}\right]$$

With the above set of potential energy parameters, Qin et al. [101] constructed an all-atomic GA model in MD simulations by mimicking the process of GA synthesis by CVD. Based on the model, they calculated the basic mechanical properties, such as Young’s modulus, buckling modulus, tensile strength, and compressive strength, under different materials parameters. In addition, they concluded a scaling principle that can be used to guide the design and prediction of the mechanical properties of pristine 3D graphene structures. In general, they found the strength and modulus scale with the density of the materials. This group also constructed 3D-printed models with surface topologies by using this principle and studied the thermomechanical properties of GAs [102]. Their simulated results show a potential relationship found among the dispersed defects, density, and thermal conductivity. Recently, MD models of both GA and hydrogel were built by Ma et al. [103] as the comparison measure for their experiments. This study mainly focused on graphene hydrogel. They found a higher mechanical strength and free-shapeable plasticity of graphene hydrogel compared with the GA. The results may indicate a potential effect of the internal medium on the mechanical properties of the GA. Tang et al. [80] adopted the GA model of Qin et al. [101] to explore the atomic deformation mechanism of porous graphene materials. The deformation behaviors, such as sliding, bending, buckling, collapsing, and densification, were observed in this model. The directly proportional relationship between material modulus and density was also verified through their simulation study.

Unlike previous studies, Patil et al. [104] conducted a more detailed study of the mechanical properties and behavior of a GA material. They optimized the model based on Qin’s work [101] by introducing the longer-range (Lennard-Jones) contributions. This allows the model to provide an accurate simulation of the highly repulsive force under compression tests. With the help of this model, the mechanical properties and fracture behavior of the GA were simulated using a variety of test methods, and the effects of material density on material properties were analyzed. In tensile testing, the tensile strength and modulus of a material significantly depend on the density of the material. The obtained correlation between tensile properties and material density [104] is in good agreement with the results reported in the literature [89,90,91,105]. In compression simulations, the density of the material affects the densification of the material and thus also significantly alters the compression properties. The fracture behavior of the material was also investigated in this work through cyclic compression tests. They found that the fracture strength of the GA decreases with the increasing length-to-height ratio of pre-existing cracks during the fracture of the GA model. The fracture toughness, on the other hand, was not related to the size of pre-existing cracks, but was shown to be related to the density of the GA material. These findings may give us a better understanding of the choice of GA materials for different applications. Later, Patil et al. [104,106] were inspired by a work of Liu’s group [107] and explored the possibility of GAs as energy absorbers. In general, porous materials have a good energy absorption capacity and GAs have shown high compressive strength and high resilience based on several experiments in the literature. They studied the shock response of GAs with the AAMD models created by Qin et al. The density of the GA materials and particle velocity were also discussed as variables, which affect the microstructural characteristics of porous models. They found that GAs can withstand general shocks. Moreover, at low densities, GAs exhibit properties like those of silica aerogels, which are already widely utilized [106]. 

The works of Patil et al. [106] were focused on testing the mechanical properties of GAs, while Zheng et al. [108] were curious about the improvement of the model structure in previous works [101]. They built a Gaussian process metamodel with a bottom-up atomistic modeling strategy to predict the mechanical properties under a certain level of stochasticity [108]. Like the models built by Qin et al. and Patil et al. [101,104,106], Zheng’s group created a simulation domain containing a random distribution of graphene sheets and spherical inclusions [108]. This model, however, sets the mechanical properties of GAs as a function of the density, and therefore the density, elastic modulus, and ultimate tensile strength are the functions of inclusion size. The inclusion size, therefore, is the only parameter studied that determines the microstructure and mechanical properties of the GAs. The calculation principle is using probability interpolation method to obtain the weighted average value of a known parameter in a function, thus calculating the value of the function. With this metamodel, the prediction of confidence intervals for the material properties and data collection can be calculated at low experimental and computational time costs.

The all-atom molecular dynamics approach to studying the mechanical properties of GAs is not limited to the simulation of experimental structures and production. Researchers have also attempted to use a top-down study strategy to find optimized structural features and to regulate the synthesis process of GAs accordingly, such as biomimetic and structure topology. 

The work of Yang et al. [12] inspired Morris et al. [109] to use molecular dynamics to simulate the mechanical behavior of a biomimetic GA. As with the *Thalia dealbata* stem, the model has a lamellar superstructure that can withstand cyclic compression without apparent permanent deformation. The aerogel with a random structure usually has inelastic deformation and poor resilience because of the weak connection in the stochastic porous structure. In contrast, the biomimetic GA has exceptional elastic properties, agreeing with previous work. Morris et al. [109] found that the determining factor of the incredible mechanical properties of the biomimetic GA is the length of the bridging structure between the two graphene sheets. This is mainly because the bridge length significantly affects the overall morphology of the model structure, which in turn determines the mechanical properties such as the elastic modulus. Moreover, they found that the anisotropic geometry of the structure of this GA material shows highly anisotropic mechanical properties under tensile and compression tests, especially in the latter. 

The structure topology of the 3D graphene network has been extensively studied in recent years [110,111,112]. The topological structure of GAs, on the other hand, has been less studied and was first mentioned in the work of Qin et al. [101]. They combined computational modeling with model experiments and applied the Schwarz surface in a 3D print model for mechanical testing. Qin’s co-workers have further investigated the topological structure model in their previous work. Based on the previous triply periodic minimal surface model of 3D graphene [101,113], Jung et al. [114] investigated the effects of structure and size on the mechanical properties of GAs. This study shows that the elastic properties and the failure mechanism under tension or shear loading are apparently affected by the cell types. Among the three surface topologies, the gyroid type shows the best mechanical performance. Lei et al. [115] also studied the microstructure of GAs based on Schwarz-surface-like graphene (SSG). They created a set of discontinuously curved GA models and measured the mechanical properties of these models in simulations. After comparing with continuous models, they found that Young’s modulus and the compression recovery ability of discontinuous models are lower than those of continuous models. This work conclusively demonstrates that enhancing the structural continuity can significantly improve the mechanical properties of GAs. Besides, a shear-strengthening phenomenon found in the G-type of SSG indicates one way in which nanostructures can tune the mechanical properties of GA materials [115].

**Figure 6:**
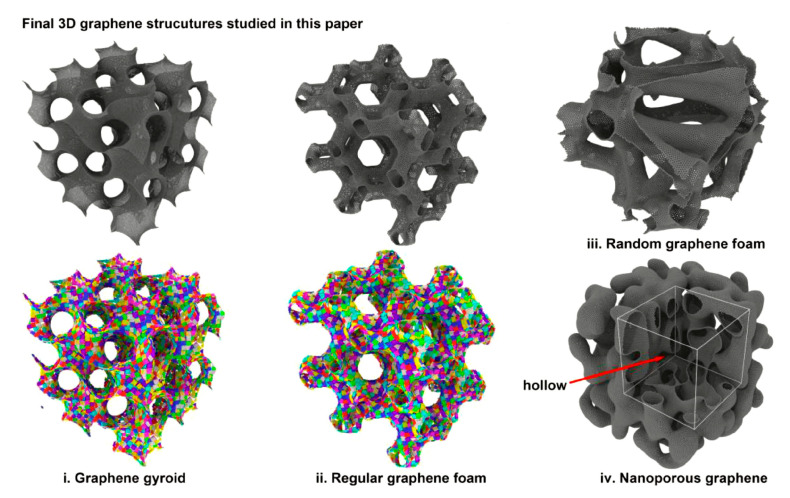
Morphological 3D GA structures studied in the paper [116].

Peng et al. [116] were also concerned with the structural topology of GAs, as shown in Figure 6, and focused on the GA structures manufactured by the template-based CVD method using all-atom molecular dynamics simulations. The template morphologies selected by researchers include gyroids, regular and random open-cell foams, and nano-porous structures. Based on these, they also set the density, surface-to-volume ratio, and deposited graphene layers as the research variables to screen out the key features of the mechanical properties of GAs. In terms of the mechanical properties of these GA microstructures, the material model with a regular structure exhibits the greatest stiffness and strength, while the non-regular model has the worst mechanical properties. For the gyroid graphene microstructure, they found a special self-stiffness phenomenon under plane stress, which enhanced the ductility and strength of the GA material. Moreover, the effects of the number of graphene layers on the mechanical properties of this structure have also been discussed. The additional van der Waals forces resulting from the increased number of graphene layers enhance the ductility of the gyroid model. However, at the same relative density, the models with fewer layers have better mechanical properties.

#### 3.1.2. Coarse-Grained Molecular Dynamics Simulation

The properties and nanostructure can be accurately reproduced by the AAMD simulations. However, the simulation scales of this method are limited by computational power and simulation costs. Therefore, most of the previously mentioned research works have used various approaches to simplify the models and reduce the computational cost, such as using individual cell units as the subject of study [117]. Based on these issues Coarse-Grained Molecular Dynamics (CGMD) methods, which are widely used in protein research, have also been gradually applied to the study of GA materials. 

The main challenge of this approach is the coarse-grained (CG) modeling, including the simplification strategy and the setting of the force field, which determines the credibility and similarity of the CG model simulations [118,119]. In CGMD simulations of GAs, the focus is on the CG modeling of graphene sheets and then on the appropriate 3D treatments of these graphene sheets. The key step is mapping the atoms of graphene sheets to the CG beads. Several studies about coarse granulation of graphene flakes and some related studies of GAs based on these studies are described below.

#### 3.1.3. Coarse-grained Model with Rectangular Mapping Strategy

To accurately describe the behavior at the nanoscale or mesoscale, a mesoscale graphene model was developed by Cranford and Buehler [120] with the hierarchical multiscale modeling strategy. This model was later widely applied in the research works of Wang’s group and his collaborators [121,122,123,124,125,126] related to the MD simulations of graphene foams (which are a type of GA material). In the CG model of Cranford and Buehler, a 25 Å × 25 Å monolayer graphene sheet is mapped into one CG bead. The CG graphene sheet model can be obtained by arranging these beads in a graphene structure. This structure of the CG graphene sheet retains some structural features of the graphene while the simulation scale is expanded to allow for mesoscale simulations.

**Figure 7:**
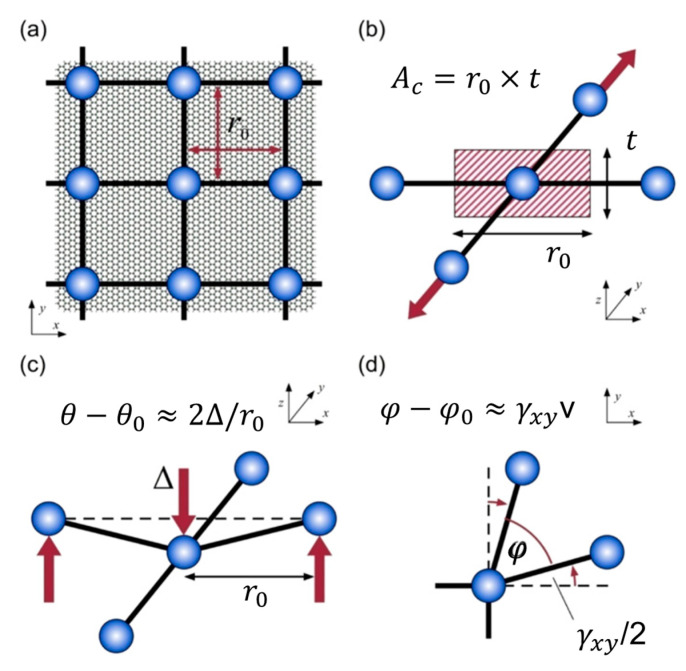
Schematic of the coarse-grain graphene model and the derived mechanical potentials [120]. (**a**) Square lattice model; each coarse-grain particle representative of 25 Å × 25 Å planar section of graphene sheet. (**b**) Geometric parameters for tensile stretching (bond) potential; associated cross-sectional area (Ac) equal to particle spacing (r_0_) by sheet thickness (t). (**c**) Geometric parameters for out-of-plane bending (one-way); change in angle between defined particle triples, θ *−* θ_0_, approximated by three-point bending. Each particle associated with two bending potentials (x- and y-direction). (**d**) Geometric parameters for in-plane distortion (shear); deviation from normal (90◦) configuration, *φ − φ_0_*, equal to shear strain, γ_xy_. Each particle is associated with four shear potentials.

The force-field potential, which is calculated based on the energy conservation principle and AAMD simulation, can be divided into bond energy, angle energy, and paired van der Waals (vdW) interactions. The total energy of the simulation system can be calculated by the sum of the energy generated by uniaxial stretching, shear deformation, out-of-plane bending, and weak interlayer interactions. The schematic of the CG graphene model and the mechanical potentials are shown in Figure 7. 

**Figure 8:**
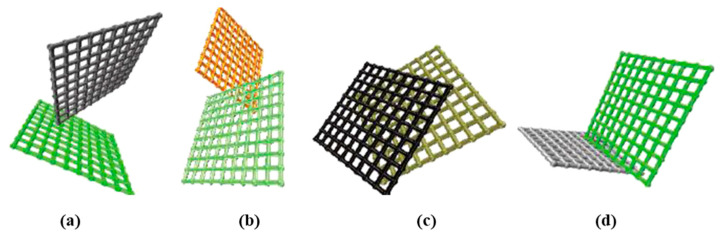
The configuration of cross-linking of CG graphene flake layers [121]. (**a**) point-surface; (**b**) edge-edge; (**c**) surface-surface; (**d**) edge-surface.

This CG graphene sheet model can well describe uniaxial tension/compression deformation, shear deformation, and out-plane deformation. Therefore, Wang et al. [121] tested the mechanical properties of the graphene foam structure model under uniaxial tension based on the model. Like the AAMD method, they simulated the production process of the material by processing randomly placed graphene sheets. A CGMD model of the materials was built after a complex system setup and assembly process. In this paper [121], they have observed and distinguished the types of microstructural contacts in the graphene foam structured materials: point-surface, edge-edge, surface-surface, and edge-surface, as shown in Figure 8a–d. Microstructural reorganization can also be identified from the four types, including in-plane and out-of-plane rotation, in-plane and out-of-plane bending and buckling, self-folding, sliding, and separation.

Wang et al. [121] revealed the microstructure deformation mechanism of graphene foam materials. They recorded the proportions and morphology of microstructures, and then calculated the constitutive relation of the model under uniaxial tension. They found three stages in the tensile stress-strain curve and analyzed the microstructure of the corresponding stage. The first stage is the linear elastic stage, the strength is determined by the elastic deformation of these microstructures. In the second stage, also the strain hardening stage, due to the rearrangement of the microstructures, such as the bending and self-folding, the material model undergoes a significant deformation but shows a worse bearing capacity. The third stage is called the necking stage in which the load capacity increases due to the compaction of the microstructures caused by the very large strain.

In addition, they also discussed the effects of the wall thickness of the model on the microscopic deformation [121]. They found a significant difference in mechanical properties between the models composed of single-layer graphene or multilayer graphene. In the actual experiment, the cell wall of graphene foam materials is made up of sheets with multilayer graphene. Wang’s group also investigated the properties of graphene foam models with two different cell wall thicknesses of graphene sheets [127]. In their study, the elastic modulus of the graphene foam increases linearly with the increasing proportion of thicker graphene sheets, while the strength first decreases and then increases as the proportion of thicker sheets increases.

Pan et al. [122] employed the model built by Cranford et al. [120] to study the tensile and fracture behaviors of mesoscopic GAs. Their model is similar to the model in Wang’s study [121] but their cell walls are made of 8-layer graphene sheets. Pan et al. [122] used a simulation approach to reproduce the experiments done by Nieto et al. [105] and tried to interpret and analyze some experimental phenomena using the CGMD method. The constitutive relation in the uniaxial tension experiment was well reproduced, including the multipeak in the stress-strain curve, ductile fracture angled at 45 degrees to the direction of tension, and the fracture surface morphology. These phenomena can be well explained by the stress distribution state and evolution of some microstructures, such as the debonding process of graphene sheets and the breaking of bonds and crosslinks. 

**Figure 9:**
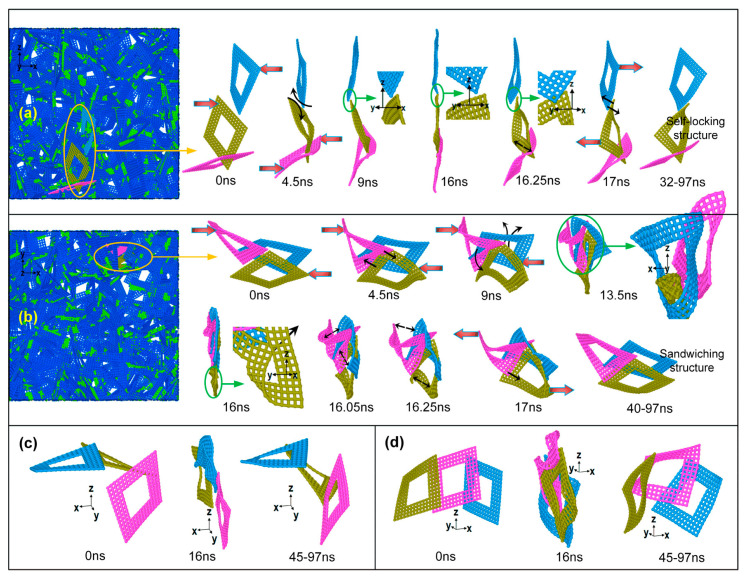
The self-locking behaviors of the microstructure investigated by the CG model [123]. (**a**) Forming process of self-locking structure (within yellow circle) in front view along the y-axis; (**b**) Forming process of the sandwiching structure (within yellow circle) in front view along the z-axis; (**c**,**d**) Two typical types of hole-induced mechanical interlocking in initial loading, super-compressive and long holding states, respectively. The fat-red and thin-black arrows indicate the external and interior forces, respectively.

Pan et al. [123] further studied the mechanical properties and microscopic mechanisms of graphene foam materials. They introduced a hole into the graphene flake as the defect and then studied the super-compression and recovery behavior of this type of GA materials using the CG model. They monitored the evolution of the microstructure in the mechanical tests and concluded that, in addition to vdW forces, mechanical interlocking between graphene sheets is the main cause of the stress residuals. The effects of graphene defects on the self-locking behaviors of the microstructure and two typical graphene interlocking configurations are both shown in Figure 9. 

**Figure 10:**
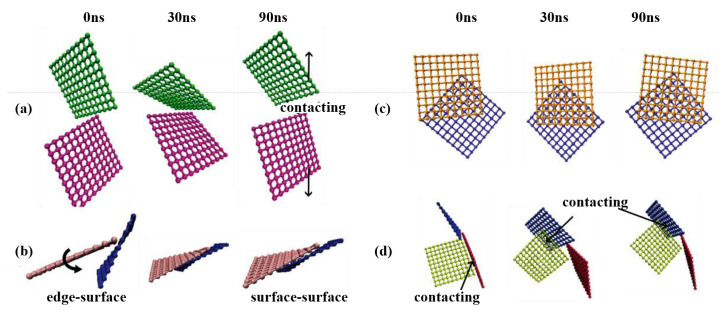
Four types of microstructural evolution in graphene foam materials [124]. (**a**) The departure of two contacting flakes; (**b**) Transformation from edge-surface contacting configuration to the surface-surface one. (**c**) the variation of contacting areas between two surface-surface contacting flakes. (**d**) The change of contacting partners.

**Figure 11:**
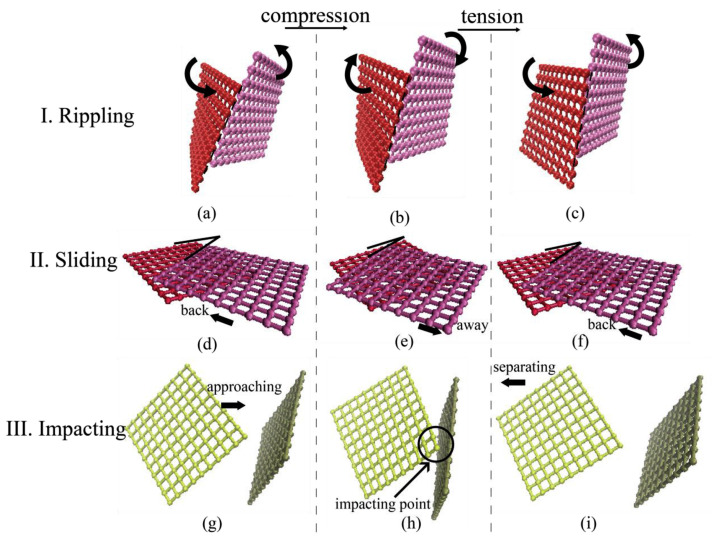
Three types of energy dissipation mechanisms of graphene foams [124]. (**a**–**c**) rippling, (**d**–**f**) sliding and (**g**–**i**) impacting.

In previous experimental research, the dissipation capacity of graphene foam materials has been well confirmed by different mechanical tests. However, the intrinsic links between the microstructures and the dissipative behaviors of this type of material are still unclear. Wang et al. [124] systematically investigated the energy dissipation mechanisms of graphene foam materials. The constitutive relation of this type of materials under different loading types was obtained based on the previously developed CG model [121,122] and experimental research [128,129]. They compared the microstructural configurations in the initial state, at maximum strain, and at unloading [124]. As shown in Figure 10, four typical ways of structural evolution were discovered, including the separation of contact sheets, the change of contact configuration, the increase of contact area, and the change of contact objects. Based on the microstructural evolution mechanism, the trends in the stress-strain curves under different loading forms were analyzed. Three energy dissipation forms, including ripping (Figure 11a–c) [129], sliding (Figure 11d–f) [130], and impacting (Figure 11g–i), have been proved in the CG graphene foam model. Several factors influencing dissipative capacity were also discussed. The study found that the energy dissipation in the first loading cycle is much larger than in later cycles, and with the increase of loading rate, the dissipation is also increased. 

**Figure 12:**
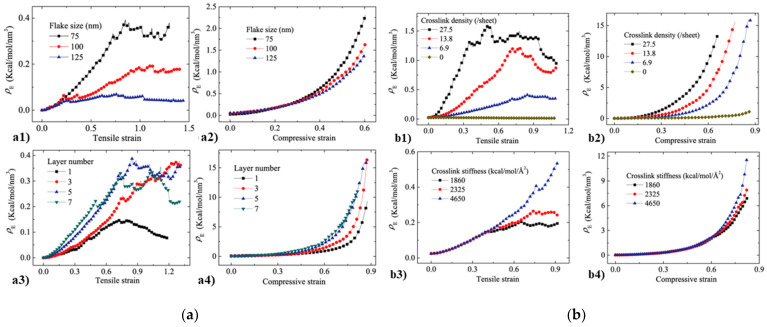
(**a**) The effects of (**a1**,**a2**) graphene flake sizes and (**a3**,**a4**) graphene flake layers of the cell walls on the elastic energy density of graphene foams; (**b**) The effects of the (**b1**,**b2**) crosslink density and (**b3**,**b4**) stiffness of the cell walls on the elastic energy density of graphene foams under both tension and compression [125].

As the most important mechanical property of porous materials in several applications, the relationship between the elasticity and microstructures of graphene foam materials is still unclear with the available experimental measurement techniques. The elastic properties and relevant mechanisms of the graphene foam materials were discussed in another work by Wang et al. [125] based on their CG model. They analyzed different deformation energies in the simulation system with different loading types and found that the bending energy dominates, which implies that bending is the dominant deformation mechanism. Besides, they summarized four potential factors affecting the elasticity of GA: the size of the graphene sheet, the number of layers of the graphene sheet, the inter-sheet crosslinking density, and the stiffness. Similar conclusions have been obtained in previous experimental studies [30,65,73]. Based on CGMD simulations, as shown in Figure 12a, Wang et al. found that GA materials with smaller cell walls and larger wall thicknesses had higher elastic strength, which was more evident in the tensile test. Crosslink density and strength also influence the elastic behavior of the GA materials to varying degrees. As shown in Figure 12b, adding more crosslinks can significantly improve the elastic behavior of the GA materials at different strains. In contrast, the crosslink strength only shows an enhancing effect at large strains. In general, making graphene foam materials with proper graphene sheet thickness of the cell walls and more physical crosslinks may be the most effective design direction to enhance their elastic properties.

#### 3.1.4. Coarse-Grained Model with Four-to-One (4-1) Hexagonal Mapping Strategy

**Figure 13:**
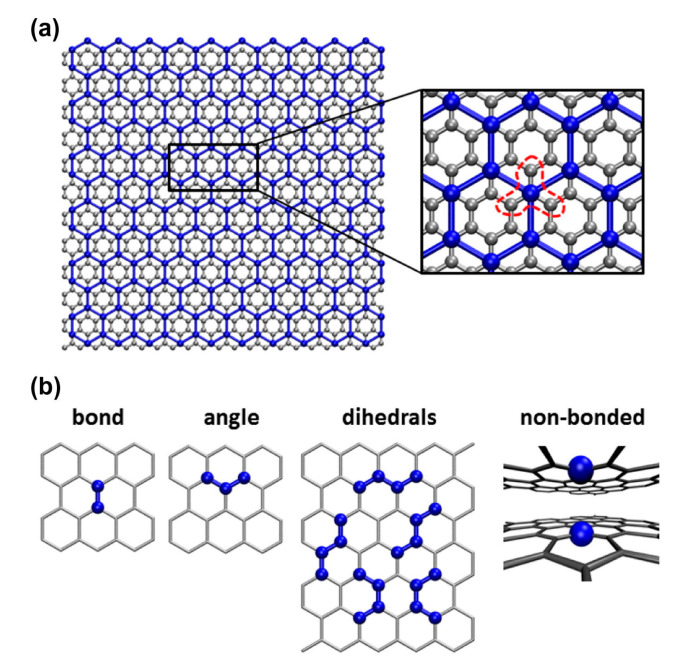
Schematic of the method of constructing CG graphene sheets [131]. (**a**) Coarse-grained lattice (blue) overlaid over the atomistic structure (grey); (**b**) Illustration of the contributions of the CG force field to the potential energy of the system.

Although the graphene flake model built by Cranford et al. [120] has considerable advantages in terms of simulation scale, it neglects many of the structural features of graphene, such as the chirality structure. Therefore, graphene foam models constructed with this CG graphene flake have difficulty in accurately capturing some of the complex mechanical behaviors, such as anisotropy of the mechanical properties, and nonlinear elasticity. Ruiz et al. [131] employed another CG strategy for the graphene sheets, a four-to-one hexagonal mapping strategy. The simplified method is based on the hexagonal lattice structure of graphene and mapping the four closest carbon atoms to one bead. The schematic of the CG model is shown in Figure 13. The force field in this model can be described by four energies: bond, angle, dihedral angle, and paired non-bonded interactions based on the energy conservation principle. 

Shen et al. [132] developed a 3D graphene model based on this CG graphene flake model, which does not consider the cross-linking of graphene sheets. The graphene foam materials with a range size of graphene sheets were tested under uniaxial compression. The results and the evolution of microstructures indicated that the stacking phenomenon tends to occur in the graphene foam material model packed with small-sized graphene sheets, resulting in a higher density and lower pore size. Under uniaxial compression, a constitutive relation with three stages similar to the previous work [121] is observed in the simulation test results for the graphene foam material model filled with small-sized graphene sheets. For the graphene foam material model having a loose filling with large-scale graphene flakes, non-linear deformation, and hardening phases are evident in the stress-strain curve, while the yielding phase is not obvious. 

Bao et al. [133] developed a graphene foam model to investigate and analyze the different influences of the size and thickness of graphene sheets on the mechanical properties of graphene foam materials. Their work indicated that the extremely low out-of-plane mechanical properties of monolayer graphene contribute significantly to the stiffness of the graphene foam materials. This is due to the susceptibility of the monolayer graphene sheets to bending or wrinkling under load, leading to the entanglement among graphene layers. This contact behavior will reinforce the interaction among monolayer graphene sheets, ultimately showing up in the higher modulus of elasticity and strength of the single-layer graphene foam material. Interestingly, this conclusion is not consistent with the work of Liu et al. [127]. This may be due to the differences in the experimental models, or there may be other influencing factors that have not been considered correctly. For the size effects of graphene flakes, Bao et al. [133] adopted the graphene sheets with a Gaussian distributed size in their graphene foam model and investigated the effects of graphene size on graphene foam material properties. The result shows that the model constructed from inhomogeneous graphene sheets has a denser network structure. Consequently, models with non-uniformly sized infilling sheets have better mechanical properties than those with uniform sizes of graphene sheets.

In addition to models developed by Ruiz et al. [131], Shang et al. [134] also built a CG model using the four-to-one mapping strategy. However, they simplified the force field of the previous model so that only the beads' coordinates and distance are needed to describe the force field of their model. The honeycomb GA model was developed based on this graphene model. Then, they studied the microstructure deformation mechanism of the GA materials during uniaxial tension, compression, and recovery. They found that the graphene foam material with multilayer graphene displayed apparent stability under compression. The uniform arrangement of graphene flakes also caused high stability but may result in reduced stress.

#### 3.1.5. Other Relevant Models

**Figure 14:**
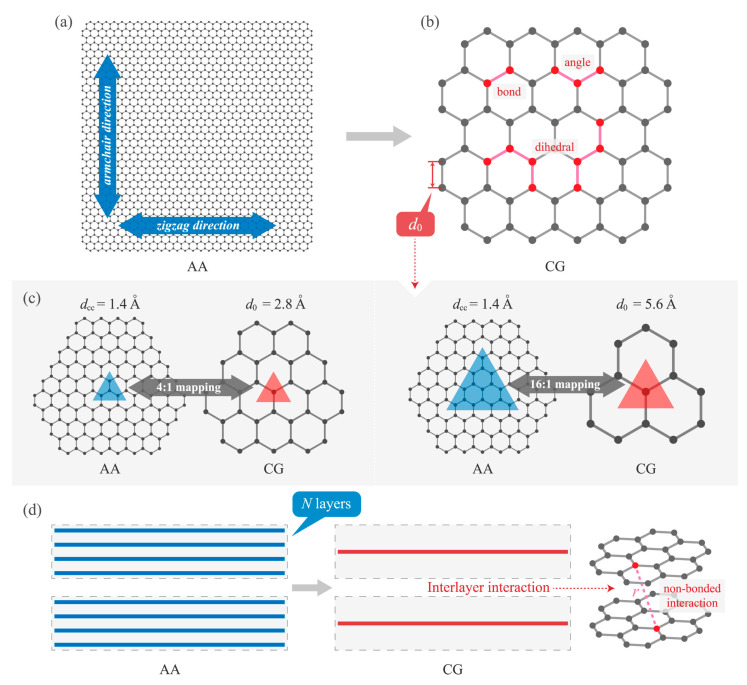
Schematics of the new CG model [135]. (**a**) All-atom model of graphene; (**b**) Corresponding CG model; (**c**) two mapping strategies; (**d**) Illustration of the coarse-graining approach applied in the thickness direction where a CG layer represents N all-atom layers.

Here are also some mapping strategies apart from rectangular mapping and 4-1 mapping methods. Wang et al. [99] constructed a 16-1 mapping strategy based on the 4-1 mapping approach. This method of mapping four CG beads onto one large bead works similarly to the 4-1 mapping model. However, various simplified strategies were analyzed and compared in the work of Liu et al. [135]. They found that the relative accuracy of models using 16-1 mapping and rectangular mapping approaches was low as shown in Table 2. Besides, they proposed a new CG strategy that not only maps the inter-plane graphene atoms to one bead, but also maps multiple layers of graphene atoms in the thickness direction to the CG bead, as shown in Figure 14. They adopted the Mie potential to accurately describe the force field of the corresponding graphene model. Compared with other models, this model exhibits high relative accuracy and enables large-scale simulation, which may be useful for future molecular dynamics modeling of GA materials and the associated simulations.

### 3.2. Finite Element Method

**Figure 15:**
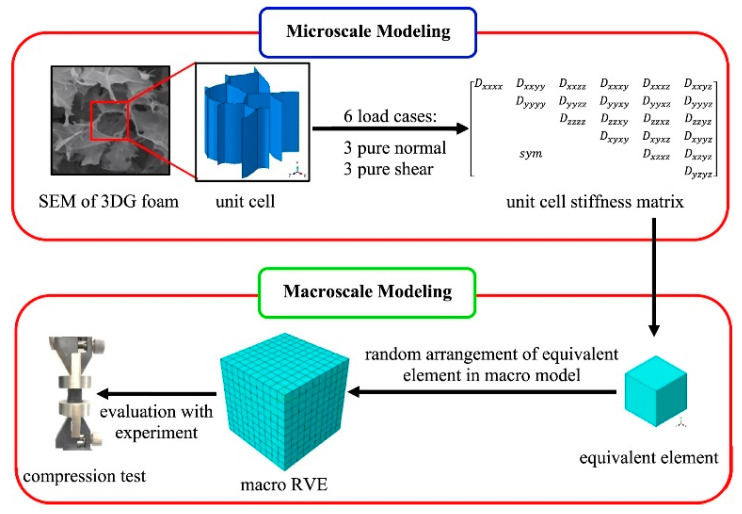
Finite element method to simulate the working mechanisms of GA materials [140].

The size of the MD simulation domain has been significantly increased with the CG method. However, the dimensions of most MD simulation models are still much smaller than the scale of the actual materials. Not only the overall size of the model, but also the size of the graphene sheets making up the model, is much smaller than the actual size. As found in previous studies on the size effects on material properties [132,133], the adoption of the small-sized graphene sheets causes an over-density of the MD model for graphene foam materials, and their mechanical properties can thus be overestimated. Therefore, Mahdavi et al. [140] proposed a new multiscale finite element (FE) model that not only considers the structural features in the microscale but also simulates the overall macroscale model (Figure 15). In their work, a unit cell with a common structure was constructed from Scanning Electron Microscopy images first. The elastic properties of this structure were then obtained from numerical simulations under different loading cases. After that, the elastic properties of the unit cell were used to represent the material structure at the microscopic scale and the equivalent elements for the construction of the macroscopic model. By defining a representative volume element that contains sufficient equivalent elements, a macroscopic model was constructed for elastic modulus estimation of graphene foam materials.

The predicted results using the multiscale FE model show smaller deviations from experimental results than those obtained from other methods, indicating that this method is a good tool for predicting the macroscopic mechanical properties of GA materials. However, the applicability of this model is limited by the homogeneity of the existing model structure. In addition, this approach is also weak in exploring the link between GA microstructure and its mechanical properties. Therefore, this method still needs further research and development.

**Figure 16:**
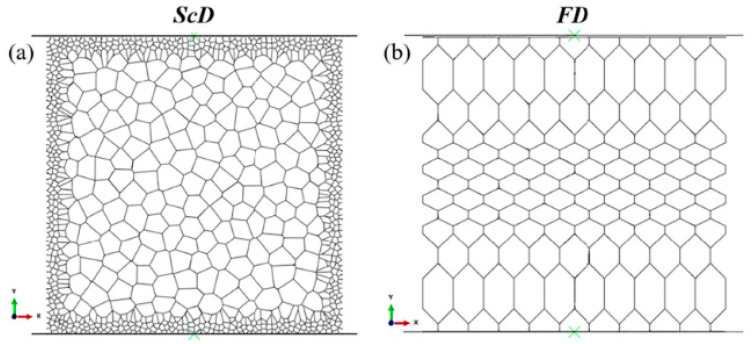
The simplified finite element models were developed by two drying methods for GA materials [76]. (**a**) GA prepared by ScD method; (**b**) GA prepared by FD method.

The finite element method (FEM) can also be used as a secondary means to obtain the mechanical properties of the microstructured GA materials. Xie et al. [76] developed two simplified FE models, as shown in Figure 16, to explore the deformation mechanisms of the microstructures prepared by two drying methods. Gao et al. [86] adopted the FEM to analyze the origin of the superior recoverability of biomimetic GA structure.

It is worth noting that the FE models are constructed by highly simplifying the actual material structure of GAs and that the scale of the models is different from the real material. Therefore, these models can only be used as an aid for most studies. 

## 4. Challenge and Outlook

Previous studies on the mechanical properties of GAs have made a positive impact on the applications of the materials, but there are still many challenges before the GA materials can be truly applied on a large scale. Firstly, in terms of experimental studies, existing production strategies of GAs are always difficult to address some problems, such as material defects, inhomogeneous quality, and parameter control. These issues make the results of experimental studies on the material parameters highly variable, resulting in a lack of sufficient reliability in the conclusions based on the experimental tests. Therefore, it is still necessary to develop new production strategies to obtain higher quality, more accurate, and controllable GAs. In addition, existing experimental studies do not have suitable criteria to evaluate the mechanical properties of GAs. As a type of multifunctional material, GAs need to be evaluated and studied for their mechanical properties while also considering other properties of the materials. The reason for the issue is that optimizing the mechanical properties may affect the structural characteristics and properties required for electrical or thermal properties. For example, as the density of a GA increases, its mechanical properties are enhanced, but its porosity decreases, which affects the electrical or adsorption properties of the GA [81]. Finally, experimental methods are limited by experimental observation techniques and equipment, which makes it difficult to capture information on some processes, such as stresses during deformation, and evolution of the material microstructure. Therefore, simulation and calculation methods are necessary to research tools to understand the mechanical properties of GAs.

However, there are some problems and challenges in the simulation methods used to study GA materials. Discussions based on simulation methods are often about ideal material models and can therefore overestimate the material properties. Therefore, It is necessary to quantify the effects of defects and inhomogeneities on GA characteristics and to improve the accuracy of simulations by introducing appropriate correction factors. In addition, the scales of the GA models created by existing simulation methods are significantly different from that of the real structure of the GAs. The experimentally measured densities of the GAs tested in the research literature are small and generally range from 3 to 20 mg·cm^−3^ [38,65,86]. However, as shown in Table 3, the densities of the GA models built by the MD method range from roughly 0.3 to 2 g·cm^−3^, which are much larger than those of the actual GA materials. This is because the modeling of the MD approach is based on microscopic particles, which requires excessive computational resources for simulations on large spatial and temporal scales. Both the AAMD and CGMD models were thus artificially reduced in scale to reduce the size of the GA cells, making the simulations cost-effective. Mahdavi et al. [140] took a multiscale FEM approach to predict the mechanical properties of some GAs and obtained relatively accurate results (deviation of about 25%). However, the model of Mahdavi et al. [140] still has some problems that affect the accuracy of the simulations and also limit the use of the model in other GAs. In addition, this multiscale simulation method is not yet able to simulate and analyze the deformation behaviors of GAs. Therefore, it is necessary and worthwhile to develop more suitable multi-scale simulation models for GA materials.

## 5. Conclusions

This review focuses on the latest studies on the mechanical properties of GA materials in terms of both experimental studies and simulation methods. Numerous experiments have proven that the mechanical properties of GAs are significantly influenced by the intrinsic properties of graphene sheets, the microstructural parameters, and the structural design. Large-sized graphene flakes can form continuous and tight graphene wall structures, thus reducing defects in the cell walls of GAs. By removing weak links between the graphene layers or introducing strong cross-linking, the interfacial adhesion between the flakes can be improved, thus allowing the cell walls to withstand large compressive deformations without collapse. The density changes due to the variations in wall thickness and pore size can also greatly affect the mechanical properties of the cell walls. Moreover, the special microstructure can optimize the deformation behavior of GAs, allowing the load to be shared more evenly and thus reducing stress concentrations. Therefore, the design and construction of an appropriate GA structure can also improve the mechanical properties of the material such as strength, stiffness, and deformability. 

In the simulation studies, the effects of the cell wall thickness, density, and structural design on the mechanical properties of GAs are also demonstrated. In addition, by simulating the deformation evolution of the GA microstructure, mechanistic issues such as behaviors and energy dissipation patterns of the GAs are also explained. In general, the existing studies have explored the intrinsic links between the microstructure and the mechanical properties of GAs in a variety of ways that can guide the design and production of GA materials.

It should be noted that there are significant limitations to the enhancement strategies for the mechanical properties of GAs due to some inherent problems in the experimental materials and simulation models. Thus, the application of GA materials still faces many challenges. Researchers need to develop new production strategies to obtain higher quality and more controllable GA materials. On the other hand, it is also necessary to develop multi-scale simulation tools to assist in the prediction, design, and analysis of the mechanical properties of GA materials, which will be an important research topic for a long time.

## Figures and Tables

**Table 1:** The mechanical properties of graphene aerogels and other aerogels.

Materials	Synthesis Methods	Density (mg·cm^−1^)	Compression		Reference
			Strain (%)	Stress (kPa)	
GA	Hydrothermal	5.1	80	18	[6]
GA	Solvothermal	3	90	5	[7]
GA	Solvothermal	1.15	90	90	[10]
GA	3D printing	123	90	1200	[9]
GA	Hydrothermal	8.3	93	50	[8]
GA	Chemical reduction	16	90	17	[57]
GA	Hydrothermal	10	95	28	[58]
GA	Hydrothermal	8	99	700	[59]
GA	Hydrothermal	6	99	1000	[11]
GA	In-situ assembly	<3	84	14.8	[60]
GA	freeze casting	8.8	92	134.1	[12]
GA	freeze casting	-	92	38	[30]
GA	Hydrothermal	5.8~7.5	99.8	0.73 × 10^6^	[61]
GA	Hydrothermal (template-based)	2.2	99	87.5	[14]
GA	In-situ assembly	3.0 to 6.3	97	4.7 × 10^3^	[62]
GA	CVD	11~70	-	-	[63]
GA	Crosslink	17.7	-	43	[27]
GA	Hydrothermal	5.07	-	5.4	[25]
GA	Hydrothermal	35.25	-	-	[64]
GA	Hydrothermal	6~8	92	4.5 × 10^6^	[65]
GA	Chemical reduction	3.7	99	20	[66]
Al aerogel	-	17.7	-	43	[67]
SiO_2_ aerogel	-	35.25	-	-	[68]
CNT sponge	-	6~8	92	4.5 × 10^6^	[69]

**Table 2:** Comparison of various CG models with the all-atom model [135].

Model	Mapping Ratio	Relative Computational Efficiency *	Relative Precision	Reference
AIREBO force field	-	1	Highest	[136,137]
Martini force field	4:1	16	High	[138]
Square CG model	245.47:1	60,256.53	Low	[120]
4:1 hexagonal CG model	4:1	16	High	[131]
16:1 hexagonal CG model	16:1	256	low	[139]
Tersoff CG model	4:1	16	High	[134]
Multilayer CG model (N-1)	2.04:1	4.16	High	[135]
Multilayer CG model (N-2)	16.33:1	266.56	High	[135]
Multilayer CG model (N-3)	38.27:1	1464.23	High	[135]
Multilayer CG model (N-4)	73.47:1	5397.75	High	[135]

* The relative computational efficiency is estimated by comparing the total pair number of atoms or CG beads in a system. The pair number is given by N_Pair_ = n(n−1)/2, where n is the total number of atoms or beads in the system. The relative computational efficiency is thus equal to (Mn(Mn−1)/2)/(n(n−1)/2) ≈ M^2^, where M is the mapping ratio. (The effect of cutoff is not considered.).

**Table 3:** Comparison of the compressive moduli of GA materials obtained from different models.

Modeling Approach	Model Density (mg·cm^−3^)	Predicted Modulus (kPa)	Experimental Modulus (kPa)	Reference
AAMD	366.2	3.01 × 10^6^	-	[101]
AAMD	230	1.60 × 10^6^	-	[114]
AAMD	695	2.95 × 10^6^	-	[104]
CGMD	450	2.3 × 10^6^ (tensile)	213 (tensile)	[122]
CGMD	950~1500	0.4 × 10^6^~2 × 10^6^	-	[132]
CGMD	200	-	-	[125]
CGMD	1070–1600	0.285 × 10^6^~2.86 × 10^6^	-	[133]
FEM	5.36~11.46	6~21	6~29	[140]

## Data Availability

Data sharing not applicable.

## Abbreviation

GA	Graphene aerogel
GAs	Graphene aerogels
3D	Three dimensional
CVD	Chemical vapor deposition
MD	Molecular dynamics
GO	Graphene oxide
vdW	van der Waals
FD	Freeze drying
ScD	Supercritical CO_2_ drying
CG	Coarse-grained
MD	Molecular dynamics
AAMD	All-atom molecular dynamics
CGMD	Coarse-grained molecular dynamics
REBO	Reactive empirical bond order
AIREBO	Adaptive intermolecular reactive empirical bond order
SSG	Schwarz-surface-like graphene
FE	Finite element
FEM	Finite element method
CNT	Carbon nanotube
